# Diverse Functions of Small RNAs in Different Plant–Pathogen Communications

**DOI:** 10.3389/fmicb.2016.01552

**Published:** 2016-10-04

**Authors:** Juan Huang, Meiling Yang, Lu Lu, Xiaoming Zhang

**Affiliations:** State Key Laboratory of Integrated Management of Pest Insects and Rodents, Institute of Zoology (CAS), BeijingChina

**Keywords:** small RNA, RNA silencing, plant immunity, pathogen virulence, plant–pathogen interaction

## Abstract

RNA silencing is a conserved mechanism that utilizes small RNAs (sRNAs) to direct the regulation of gene expression at the transcriptional or post-transcriptional level. Plants utilizing RNA silencing machinery to defend pathogen infection was first identified in plant–virus interaction and later was observed in distinct plant–pathogen interactions. RNA silencing is not only responsible for suppressing RNA accumulation and movement of virus and viroid, but also facilitates plant immune responses against bacterial, oomycete, and fungal infection. Interestingly, even the same plant sRNA can perform different roles when encounters with different pathogens. On the other side, pathogens counteract by generating sRNAs that directly regulate pathogen gene expression to increase virulence or target host genes to facilitate pathogen infection. Here, we summarize the current knowledge of the characterization and biogenesis of host- and pathogen-derived sRNAs, as well as the different RNA silencing machineries that plants utilize to defend against different pathogens. The functions of these sRNAs in defense and counter-defense and their mechanisms for regulation during different plant–pathogen interactions are also discussed.

## Introduction

Small RNAs (sRNAs) are 20–30 nucleotide (nt)-long non-coding RNA molecules, which are widely present in eukaryotic organisms. It is well established that sRNAs are involved in the regulation of gene expression through a process generally termed RNA silencing. RNA silencing contributes to almost all eukaryotic cellular processes, including preventing the invasion of viruses or transgenes, inhibiting the movement of transposable elements, and regulating developmental and physiological processes (Itaya et al., 2008; Wang et al., 2011a; Castel and Martienssen, 2013; Bond and Baulcombe, 2014; Holoch and Moazed, 2015).

Plant sRNAs are divided into two major classes: microRNAs (miRNAs) and small interfering RNAs (siRNAs). Most miRNAs are 21–24 nt in length and derived from RNAs with imperfectly base-paired hairpin structures (Chen, 2009), while siRNAs are generated from perfectly complementary long dsRNAs (Xie et al., 2004). Plant siRNAs are grouped into four subclasses: *trans*-acting siRNAs (ta-siRNAs), heterochromatic siRNAs (hc-siRNAs), natural antisense transcript-derived siRNAs (nat-siRNAs), and long siRNAs (lsiRNAs). Proteins, such as Dicer-like proteins (DCLs), HYPONASTIC LEAVES 1 (HYL1), HUA ENHANCER 1 (HEN1), and Serrate (SE) are involved in sRNA biogenesis pathways (Katiyar-Agarwal and Jin, 2010; Rogers and Chen, 2013; Holoch and Moazed, 2015). Some siRNAs require RNA-dependent RNA polymerases (RDRs) and suppressor of gene silencing 3 (SGS3) for amplification (Sijen et al., 2007). After processing and amplification, sRNA duplexes are sorted and loaded into Argonaute (AGO) proteins, and the passenger strand is discarded. In animals, the passenger strand is removed by slicing or unwinding in an ATP-dependent reaction (Liu and Paroo, 2010). In plant, however, the removing mechanism of the passenger strand is still unclear. Matured RNA-induced silencing complexes (RISCs) with the guide strands anneal to its complementary sequence and regulate gene expression at transcriptional and post-transcriptional levels through DNA methylation, chromatin modification, mRNA slicing, mRNA degradation, or translational inhibition (Ghildiyal and Zamore, 2009; Zhang X. et al., 2011).

One of the important functions of RNA silencing is to suppress the infection of pathogens. The RNA silencing machinery of host plants can directly target the genomic RNA and transcripts of viruses, viroids, and virus satellites to suppress their RNA accumulation. However, plants are also susceptible to other pathogens, such as bacteria, fungi, oomycetes, and nemotodes, which unlike viruses, do not replicate or expose their genome in host cells during any part of the infection process. To defeat these pathogens, plants have evolved complicated defense systems, including PAMP-triggered immunity (PTI) and effector-triggered immunity (ETI) (Jones and Dangl, 2006). When successful pathogens evolve new effectors to suppress the host ETI response, plants respond by evolving novel resistance (R) proteins to recognize the effectors and trigger ETI responses in this endless arms race.

Both miRNAs and siRNAs contribute to PTI and ETI by fine-tuning plant hormones and/or silencing the genes involved in pathogen virulence (Navarro et al., 2006; Zhang W. et al., 2011). While host sRNAs play important roles in pathogen resistance, pathogens also encode sRNAs to manipulate host defense responses, as well as mediate pathogen virulence. sRNAs in fungi, oomycetes, and bacteria have been shown to function in promoting pathogen virulence. In fungi and oomycetes, sRNAs are mostly generated from transposable element (TE) regions (Nunes et al., 2011; Vetukuri et al., 2012; Weiberg et al., 2013). Key proteins in the RNA silencing machineries, such as DCLs, AGOs, and RDRs, are also present in these eukaryotic plant pathogens and are involved in the biogenesis and function of some sRNAs (Murata et al., 2007; Vetukuri et al., 2011). However, the biogenesis of sRNAs in fungi is more diverse than in plants. Both DCL-dependent and DCL-independent siRNA biogenesis mechanisms were identified in fungi *Neurospora crassa* (Lee et al., 2010). Furthermore, at least four different mechanisms that use distinct combinations of proteins, including Dicers, QDE-2, the exonuclease QIP, and an RNAse III domain-containing protein MRPL3, were proposed to be involved in the biogenesis of miRNA-like small RNAs (milRNAs) in *N. crassa* (Lee et al., 2010). Bacterial non-coding sRNAs are different from sRNAs in eukaryotes (Weiberg et al., 2014). They functionally associate with distinct RNA-binding protein complexes, including the clustered regularly interspaced short palindromic repeat (CRISPR)-associated (Cas) system (CRISPR-Cas) (Fahlgren et al., 2007; Li et al., 2010; Zhang X. et al., 2011; Wiedenheft et al., 2012), the RNA chaperone Hfq (Schu et al., 2015), and CsrA/RsmA (Schu et al., 2015), and regulate the expression of target mRNA through short and impacted base-pair (10–25 nt). Meanwhile, viroids, the smallest known pathogen, which does not code for proteins, have been proposed to encode specific sRNAs that target host genes and result in disease symptoms (Wang et al., 2004). Furthermore, some virus-derived siRNAs (vsiRNAs), which are generated to target viral RNAs, may target host genes, and subsequently mediate the viral disease symptom. Whether viral fitness would be increased by vsiRNAs remains unknown (Qi et al., 2009; Xia et al., 2014). Viruses, oomycetes, and bacteria have RNA silencing suppressors and other effectors that directly inhibit host sRNAs, while some fungi that localize in the intercellular space of plants deliver fungal sRNAs as effectors into plant cells to inhibit the plant PTI response. In this review we will discuss our current understanding of sRNAs in plants and plant pathogens, focusing on their functional differences in plant–pathogen interactions.

## Plants Encode sRNAs That Fine-Tune Plant Hormones and Antimicrobial Activity to Defend Against Pathogen Attack

**FIGURE 1:**
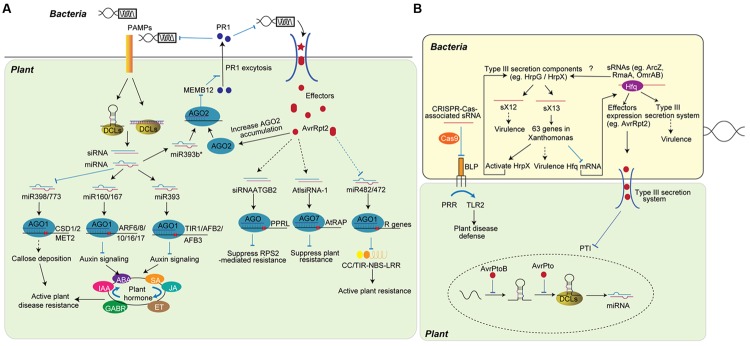
**Role of sRNAs in plant-bacteria interaction. (A)** Plant sRNAs defend bacteria attack by fine-tuning plant hormone and disease resistance activity. Upon the infection, plants detect PAMPs and modulate the accumulation of miRNA and siRNA. miRNAs, such as miR393, miR160, and miR167, regulate disease resistance by fine-tuning plant hormone networks, while other miRNAs regulate the activation of R protein (miR482/miR472) or the slicing of genes inhibiting plant immunity (miR398/miR773). miR393b*, the pairing strand of miR393, increases plant immunity by promoting the exocytosis of antimicrobial protein. siRNAs, including siRNAATGB2 and AtlsiRNA-1, are induced by bacteria effectors and enhance ETI by silencing genes that negatively regulate plant disease resistance. **(B)** Bacteria non-coding sRNAs (ncRNAs) regulate bacteria gene expression to improve virulence. Through imperfect base-pairing of short regions (10- to 25-nt), bacteria ncRNAs bind to target mRNAs and guide the suppression of genes or proteins that are involved in virulence. ncRNAs can regulate bacteria virulence by inhibiting proteins that trigger host defense (BLP) or affecting the expression of effectors (AvrRpt2). Bacteria effectors translocate into host plant cell and inhibit the regulation of plant sRNA (bottom). The AvrPtoB effector specifically represses the accumulation of miR393 at the transcriptional level, while AvrPto reduces the processing of miR393.

**FIGURE 2:**
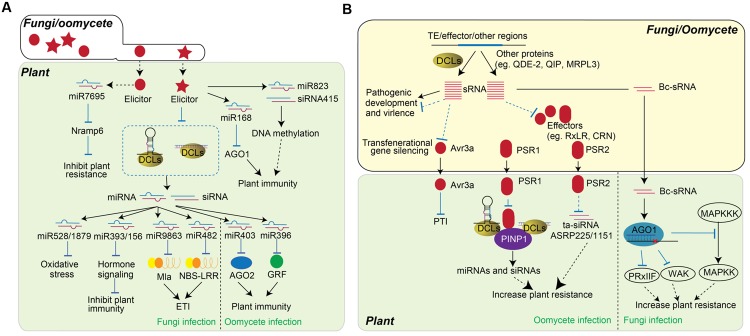
**Role of sRNAs in plant-fungi/oomycete interaction. (A)** Plant sRNAs regulate PTI and ETI in response to fungi or oomycete infection. The infection of fungi (left) and oomycete (right) alters the accumulation of miRNAs, by which changes the expression of genes contribute to plant resistance. Fungi elicitorsor fungi infections triggers the accumulation of some sRNAs, such as miR7695, miR168, miR823 and siRNA415, while miR528, miR1879, miR9863, and miR482 are down-regulated to improve plant resistance. The accumulations of miR403 and miR396 are down-regulated upon oomycete infection. **(B)** Schematic representation of the function of fungi/oomycete sRNAs in pathogen virulence. sRNAs encoded by fungi and oomycetes are usually generated from TE region, effector coding region, and other regions. These sRNA can be either DCL-dependent or DCL-independent and are involved in the regulation of pathogen development and virulence. In particular, sRNA regulate the expression of effectors, which further influence the accumulation of host miRNA and siRNA. sRNAs generated from Avr3a region of oomycete can transgenerationally change the pathogen virulence. The PSR1 and PSR2 effectors of oomycete are secreted into plant cells and alter host RNA silencing machineries as RNA silencing suppressor to decrease host immunity. On the other hand, fungi sRNAs, Bc-sRNAs, translocate into host cell and utilize plant RNA silencing component to reduce the expression of host immune genes and facilitate fungi infection.

Although a potent immune system is necessary for plants to survive pathogen infections, it also deprives the limited resources available for plant growth and development. Although more studies need to be done, a constitutively active immune system in plants may result in reduced growth and seed yield (Tian et al., 2003; Walters and Heil, 2007). Thus, plant immune responses must be tightly regulated, and one strategy is to generate endogenous sRNAs that silence specific genes involved in plant hormone production or antimicrobial activity. Upon infection, the biogenesis and/or the accumulation of these sRNAs are regulated, which subsequently fine-tune plant hormone levels and the expression of genes involved in plant resistance (**Figures 1A** and **2A**).

As far as we know, bacteria, fungi, and oomycetes infect plants without direct genome and RNA interaction with the host RNA silencing machineries. To these pathogens, fine-tuning of the plant immune system is critical for host resistance. Various plant miRNAs and siRNAs play critical roles in anti-bacterial resistance (**Figure 1A**). miR393 is the first miRNA shown to function in anti-bacterial defense. The accumulation of miR393 is up-regulated upon the treatment of the conserved N-terminal part of flagellin, flg22, or the infection of bacterial pathogen *Pseudomonas syringae* pv. *tomato* (*Pst*) DC3000. miR393 enhances host resistance to *Pst* DC3000 by negatively regulating the expression of F-box auxin receptors, including Transport Inhibitor Response 1 (TIR1), Auxin signaling F-Box proteins 2 (AFB2), and 3 (AFB3) (Navarro et al., 2006). Further studies in rice determined that miR393 is a *bona fide* stress-related miRNA that is widely involved in plant resistance to other pathogens and abiotic stresses, such as salt and drought (Bian et al., 2012; Xia et al., 2012; Campo et al., 2013). In addition to miR393, miR160 and miR167 also target Auxin response factor (ARF) family transcription factors and are induced by infection with *Pst* DC3000 *hrcC*^-^, a strain with a mutated type III secretion system, to improve plant antibacterial defense (Fahlgren et al., 2007). Further studies uncovered that miR160a and 15 other miRNAs are induced upon flg22 treatment. On the other hand, miR398b, miR773, and 9 other miRNAs are down-regulated upon flg22 treatment (Li et al., 2010). Over-expression of miR398b and miR773 attenuates PTI by repressing flg22 or bacteria-induced callose deposition, indicating miRNAs play important roles in disease resistance. However, the over-expression of miR160, which increases PAMP-induced callose deposition, did not significantly change the basal defense of plant to *Pst* DC3000 bacteria, suggesting a complicate miRNA regulatory network in plant disease responses (Li et al., 2010). Furthermore, miR393b*, the complementary strand of miR393, is loaded into AGO2 and regulates plant resistance by suppressing the expression of MEMB12. MEMB12 is a Golgi-localized SNARE protein, and its down-regulation leads to increased exocytosis of PR1, which subsequently enhances plant resistance (Zhang X. et al., 2011). Thus, miR393 and miR393b*, two sRNAs generated from a same sRNA duplex, bind AGO1 and AGO2 respectively to regulate distinct hormone pathways and coordinately increase plant immunity (Navarro et al., 2006; Zhang X. et al., 2011). Another interesting finding about miRNA in bacterial defending is that one miRNA can target both negative and positive regulators of immunity depending on the timing and the amplitude of defense responses. miR863-3p improves plant defense by silencing a typical receptor-like pseudokinase1 (ARLPK1) and ARLPK2 during early infection, and negatively regulates defense by silencing *SE* gene during later infection (Niu et al., 2016).

In response to sRNA-mediated PTI, successful pathogens deliver effectors into host cells to interfere with PTI. For detailed information about the role of pathogen effectors, several reviews are available (Dou and Zhou, 2012; Feng and Zhou, 2012). To counteract pathogen effectors, plants induce ETI. As ETI is more robust and usually triggers a hypersensitive response (HR), the ETI reaction is strictly regulated by siRNAs and miRNAs. siRNA nat-siRNAATGB2, which is specifically induced by *Pst* DC3000 effector protein AvrRpt2, enhances ETI by suppressing the expression of pentatricopeptide repeats (PPR) protein-like gene (PPRL) and preventing the negative effect of PPRL on the resistance pathway mediated by *RPS2*, a resistance gene that specifically recognizes effector AvrRpt2 (Katiyar-Agarwal et al., 2006). AtlsiRNA-1, which is also induced by AvrRpt2, improves disease resistance by silencing the expression of AtRAP, a negative regulator of plant disease resistance (Katiyar-Agarwal et al., 2007). In addition to these sRNAs, genome-wide sRNA deep sequencing indicates that the accumulation of more than 20 miRNAs and various nat-siRNAs are significantly altered upon ETI (Zhang M. et al., 2011; Zhang et al., 2012). Some targets of these miRNAs are key genes contributing to the hormone biosynthesis and signaling pathways involved in plant resistance. A TE-siRNA, TE-siR815, generated from the intron of WRKY45-1, represses ST1 and subsequently attenuates WRKY45-mediated resistance to *Xanthomonas oryzae pv. Oryzae*, which results in the opposite functions of *WRKY45-1* and *WRKY45-2* (Zhang et al., 2016).

Host sRNAs contribute to ETI not only by regulating the expression of genes involved in plant resistance but also by directly regulating the activation of R proteins. For instance, *RPP4* and *SNC1*, two *R* genes located in the *RPP5* locus, are involved in disease resistance against bacterial and fungal pathogens (Baldrich et al., 2014, 2015). A study demonstrated that these *R* genes are negatively regulated by RNA silencing. The *SNC1* gene was up-regulated in *dcl4* and *ago1* mutants (Yi and Richards, 2007). When a pathogen interferes with host RNA silencing, it may subsequently disturb the sRNA-mediated inhibition of *R* genes and activate the function of these R proteins. However, sRNAs complementary to the *SNC1* region are not increased in *dcl4* and *ago1* mutants, suggesting that other sRNAs may contribute to the up-regulation of *SNC1* in these mutants (Yi and Richards, 2007). The accumulation of miR482 is decreased in plants infected with *Pst* DC3000 but not *Pst* DC3000 *hrcC*^-^ (Shivaprasad et al., 2012). Further study predicted that miR482 can target mRNAs of 58 coiled-coil, nucleotide-binding site, leucine rich repeat proteins (CC-NBS-LRR). Meanwhile, the production of secondary siRNA, caused by the targeting in a RDR6 dependent manner, may target other mRNAs of a defense-related protein. Thus, upon the infection of virus or bacteria, the accumulation of miR482 is decreased to suppress the miR482-mediated silencing cascade, and subsequently increase the expression of defense-related mRNAs (Shivaprasad et al., 2012). miR482 in cotton was also reported to target more than 10% of NBS-LRR genes and triggers the production of secondary siRNAs. Infection with fungal pathogen *Verticillium dahliae* down-regulates miR482 accumulation and increases NBS-LRR gene expression in cotton (Zhu et al., 2013). Interestingly, *Arabidopsis* miR472, which targets RPS5 CC-NBS-LRR genes, modulates both PTI and ETI pathways. Mutation in miR472 results in increased resistance to both *Pst* DC3000 and *Pst* DC3000 *avrPphB* (Boccara et al., 2014).

Host sRNAs also regulate PTI and ETI upon various fungal and oomycete attack (**Figure 2A**). *Magnaportbe oryzae* is a rice blast fungus that causes rice blast disease. The accumulation of rice miRNA528/miR1879 is down-regulated by treatment with a *M. oryzae* elicitor, resulting in up-regulation of their target genes that control oxidative stress (Baldrich et al., 2015). Meanwhile, the accumulation of miR393b/miR156 are also negatively altered upon the elicitor treatment of *M. oryzae* on rice (Campo et al., 2013). On the other hand, a novel DCL4-processed miRNA, osa-miR7695, was identified in rice to target an alternatively spliced transcript of *Nramp6* (Natural resistance associated macrophage protein 6) gene and its overexpression results in enhanced resistance to *M. oryzae* infection (Campo et al., 2013). Further study identified a group of small RNAs, including miR156, miR165/166, miR170, and miR172 in *Arabidopsis* that were regulated by elicitors of the fungus *Fusarium oxysporum*. Particularly, miR168, which is known to regulate plant abiotic responses via control of AGO1, was transcriptionally activated, and its upregulation negatively correlated with AGO1 transcripts (Baldrich et al., 2014). In addition, miR823 and siRNA415, both of which are involved in RNA-directed DNA methylation (RdDM), were also found to be induced by fungal elicitors (Baldrich et al., 2014). The regulation of these sRNAs by fungal elicitors suggests their functions in PTI. *Puccinia graminis* f.sp. *tritici* (*Bgt*) is a powdery mildew fungus that causes devastating disease in wheat, barley, and other plants. Eight different miRNAs, miR159, miR164, miR167, miR171, miR444, miR408, miR1129, and miR1138, that regulate three different defense response processes are significantly induced at the early, but not the late, stage of *Bgt* infection. Thus, these miRNAs may play a key role in HR at the onset of disease (Gupta et al., 2012). The roles of sRNA in plant immune response were further demonstrated in powdery mildew fungus *Blumeria graminis* f. sp. *hordei* (*Bgh*) (Liu et al., 2014). *Mildew resistance locus a* (*Mla*), encoding a group of CC-NBS-LRR proteins that respond to *Bgh*, are targeted by the miRNA family miR9863. miR9863 was shown to guide the cleavage of *Mla1* transcripts in barley, and down-regulate the accumulation of MLA1 protein in the *Nicotiana benthamiana* expression system. In addition, miR9863 can trigger the biogenesis of 21-nt phased siRNAs (phasiRNAs) and further repress the expression of *Mla1*. Over-expression of miR9863 specifically attenuates Mla1-mediated cell death and disease resistance (Liu et al., 2014). miR482 in potato can also target NBS-LRR genes. *V. dahliae* infection down-regulates the accumulation of miR482, which in turn increases NBS-LRR gene expression (Yang et al., 2015). The silencing of NBS-LRR genes by these specific 22-nt miRNAs, and their activation after miRNA down-regulation upon bacteria, fungal, or viral treatments, have been widely studied in different plants (He et al., 2008; Xin et al., 2010; Zhai et al., 2011; Li et al., 2012; Shivaprasad et al., 2012; Zhu et al., 2013; Boccara et al., 2014; Liu et al., 2014; Fei et al., 2015). *Phytophthora sojae* is a notorious oomycete that infects soybean root and stem. *P. sojae* infection down-regulates the expression of miR403, a miRNA that targets AGO2, a positive regulator of plant immunity (Guo et al., 2011). Similarly, the accumulation of sRNAs and their targets are also differently regulated in susceptible and resistance soybean cultivars. The expression of miR396 in *Solanaceae* is down-regulated upon infection with another oomycete, *Phytophthora infestans*. Over-expression of miR396 resulted in the down-regulation of GRF targets and increased susceptibility to *P. infestans* (Chen et al., 2015). It is clear that plant sRNAs play a critical role in regulating the expression of genes involved in plant defense and immunity. However, each sRNA has distinct function in plant immune response, and the accumulation and the function of sRNAs are pathogen-dependent. Therefore, in order to obtain the systematic role of RNA silencing in plant resistance, the function of more sRNAs needs to be further investigated.

## Plants Utilize sRNAs to Defend Against Pathogen by Directly Targeting on Viral and Viroid Genomes and Transcripts

Viruses and viroids infect plants by replicating their genomes inside the host cells. Post-transcriptional gene silencing (PTGS) was first identified in both transgenes processing and *Potato virus X* (PVX) infection. sRNAs complementary to the sense transcript of the transgene and the positive strand of PVX were discovered, indicating that sRNAs participate in PTGS transgene silencing and viral defense (Hamilton and Baulcombe, 1999). Further studies revealed that the replication of viruses and viroids, and the folding of their RNA genomes and transcripts, produce dsRNAs that recruit RNA silencing machinery (Ding, 2009).

**FIGURE 3:**
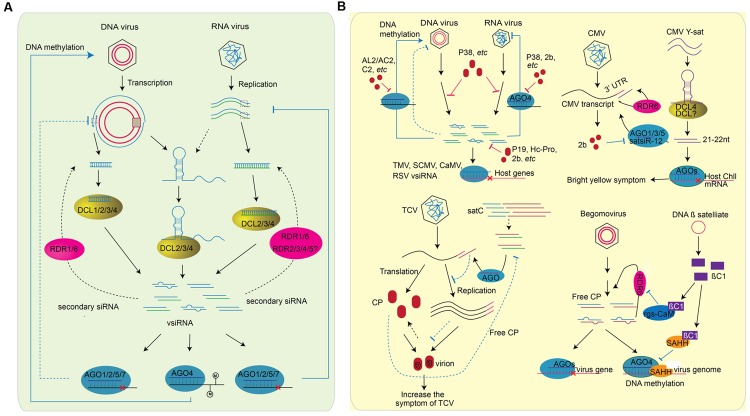
**Role of sRNAs in plant-virus interaction.** RNA silencing inside plant cells can be divided into two parts: **(A)** Plant generate vsiRNAs, targeting on virus genome directly to defend viral infection. The generation of vsiRNA are slightly different for RNA virus or DNA virus. For RNA virus, the structure region of virus genome, dsRNA replicative intermediate forms of ssRNA viruses, and the dsRNA genomes of dsRNA viruses can be processed by DCL proteins (right). The vsiRNAs of DNA virus, on the other hand, can be processed from the structured region of the transcript and the overlapping region of the bi-direction transcription (left). In both cases, RDR1 and RDR6 are involved in the generation of secondary vsiRNA (shown in blank dash line). After generation, vsiRNAs are loaded into different AGOs and perform the silencing of virus genome. vsiRNAs target on RNA virus to slice the genomic RNA, while perform DNA methylation on the genome of DNA virus. Whether vsiRNA targets on the transcription of DNA virus remains unknown (blue dash line). **(B)** The counter-defense of virus to plant RNA silencing machinery. As plant generates vsiRNA to silence virus genome, viruses encode suppressors, such as 2b, Hc-Pro, P19, AL2/AC2, P38, and etc., as a counter-defense (left above). The effect of suppressor on RNA silencing include the interfere of DCL slicing, the blocking of methylation, the binding of vsiRNsA, the preventing of RISC assembly, and etc. vsiRNAs encoded by TMV, CMV, CaMV, and RSV can also target the host genes to decrease plant defense. In addition, plant viruses are often accompanied with a variety of subviral RNA/DNAs. These satellite RNA/DNAs affect virus pathogenicity by generating satRNA-derived siRNAs (satsiRNAs). CMV Y satellite (Y-sat) produces a 22-nt satsiRNA that targets Chll, a key gene involved in chlorophyll synthesis, resulting in bright yellow symptom. sat-siR-12, another satsiRNA can loaded into AGO1/3/5 and regulate CMV transcripts accumulation with the function of RDR6. As counter defense, CMV encodes VSR 2b to inhibit the function of AGOs (right above). TCV is often accompanied with a single strand satellite RNA (satC) that is composed of the 3′ end of TCV helper virus (left bottom). Because of the sequence similarity of satsiRNA and the 3′ end of TCV helper virus, the presence of satC-siRNA represses the accumulation of TCV genomic RNA. At the same time, TCV genomic RNA and the CP protein assemble to a virion. CP is a VSR encode by TCV. The down-regulation of TCV transcripts by satC-siRNAs result in the increase of free CP protein, which subsequently suppresses the accumulation of satC-siRNAs (shown in dash line). DNA ß satellites are circular ssDNA that associate with many monopartit begomoviruses. The ßC1 protein encoded by DNAß satellite is a VSR that suppresses TGS by the interaction with SAHH, and PTGS through the interaction with rgs-CaM (right bottom).

Viruses contain either single-stranded RNA (ssRNA), double-stranded RNA (dsRNA), ssDNA, or dsDNA genomes (Ding and Voinnet, 2007). During the replication of an ssRNA viral genome, a complementary strand of RNA is synthesized, which forms a long dsRNA with the original viral genome. The dsRNA replicative intermediate forms of ssRNA viruses and the dsRNA genomes of dsRNA viruses can be targeted by host RNA silencing machineries (**Figure 3A**). Nearly equal amounts of positive and negative strand vsiRNAs without positional bias were derived from *Cucumber yellows closterovirus* (CuYV), *Turnip mosaic potyvirus* (TuMV), CMV, *Watermelon mosaic virus* (WMV), PVX, and *Tomato yellow leaf curl virus* (TYLCV), all positive ssRNA viruses from different families (Yoo et al., 2004; Ho et al., 2006; Donaire et al., 2009; Wang et al., 2010). It was also shown that vsiRNAs were nearly equally derived from the positive and negative genome of *Rice stripe virus* (RSV), an ambisense virus with four genomic ssRNAs (Yan et al., 2010). However, more than 80% of vsiRNAs derived from *Cymbidium ringspot virus* (CymRSV) are generated from the positive strand (Molnar et al., 2005). Similar phenomena are also observed in plants infected with other ssRNA viruses such as TCV, *Tobacco mosaic virus* (TMV), *Tobacco rattle virus* (TRV), and *Pepper mild mottle virus* (PMMoV), in which some positive strand vsiRNAs can account for 97% of total vsiRNAs (Ho et al., 2006; Donaire et al., 2009; Qi et al., 2009). There are no dsRNA intermediate replicative forms for ssDNA and dsDNA viruses. Some vsiRNAs generated from DNA viruses display strong strand bias, indicating that these vsiRNAs may be processed from the structured region of the viral RNA transcripts. 62% of the vsiRNAs match the transcript polarity of *Cauliflower mosaic virus* (CaMV), a virus from which the commonly used constitutive 35S promoter is derived. Although up to 82% of vsiRNAs are generated from the leader region, these exhibit no strand bias (Blevins et al., 2011). *Tomato yellow leaf curl China virus* (TYLCCNV) is a *Geminiviridae* that has an ssDNA genome. Although the vsiRNAs derived from TYLCCNV display site bias, they map nearly equally to the positive and negative genomes (Yang et al., 2011a). Thus, both the dsRNA replicative form and the secondary structure of viral genomes can processed by host RNA silencing machineries. However, the implication of these findings on viral pathogenicity or evolution is still unknown.

**FIGURE 4:**
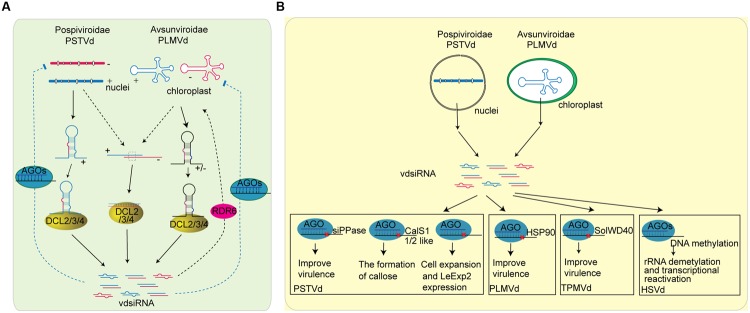
**Role of sRNAs in plant-viroid interaction. (A)** The biogenesis of vdsiRNAs in plant and the possible function of vdsiRNA in plant defense to viroid. PSTVd is mainly found in nucleolus, and its vdsiRNAs predominately map to the positive strand of the left and right terminal regions. It is most likely that PSTVd-vdsiRNAs are generated from the hairpin or stem-loop structure of plus-strand of PSTV transcripts. The secondary structure of PSTVd transcripts are targeted by DCL protein and sliced into vdsiRNA. Another possible source of vdsiRNA are the accidental association of (+) and (-) strand replication, which are further target by DCL protein. On the other hand, PLMVd, viroid that replicate in the chloroplast, generate vdsiRNAs from both polarities. The stem-loop structures of PLMVd are processed by DCL protein to generate vdsiRNAs. Furthermore, some research indicate that vdsiRNAs can be amplified through the activity of RDRs. After generation, vdsiRNA may be loaded into plant AGO proteins and target viroid RNAs. **(B)** The function of vdsiRNAs in producing viroid symptom. Some of the viroid symptom maybe caused by vdsiRNAs that target host genes. vdsiRNA generated by PSTVd can target on various plant genes including soluble inorganic pyrophosphatase (siPPase) gene, callose synthase genes CalS11-like and CalS12-like, and LeExp2 gene, while PLMVd vdsiRNA has been reported to target HSP90 and trigger signal transduction that eventually leads to viroid disease symptoms. TPMVd vdsiRNA has also been shown to slice the SolWD40 gene. In addition, HSVd vdsiRNAs are involved in TGS by inducing DNA methylation of the promoter region of rRNA genes.

Viroids, the smallest pathogen that can replicate in the nucleus or chloroplast, consists of naked, single-stranded, closed circular RNAs with sizes ranging from 250- to 400-nt (Ding, 2009). More than two decades ago, people noticed that *Potato spindle tuber viroid* (PSTVd) infection results in full methylation of the PSTVd cDNA sequence that is inserted into the tobacco genome (Wassenegger et al., 1994). This methylation occurs by viroid-induced RNA silencing and RdDM. Later studies detected siRNAs in PSTVd-infected tomato and tobacco plants and proved that viroids are the activator and target of RNA silencing (**Figure 4A**) (Itaya et al., 2001; Papaefthimiou et al., 2001). Viroid-associated siRNAs (vdsiRNAs) of PSTVd are generated from both polarities in the left and right domains. By profiling PSTVd vdsiRNAs through deep sequencing, Itaya et al. (2007) uncovered that PSTVd vdsiRNAs predominately map to the positive strand of the left and right terminal regions, indicating that these sRNAs are generated from the secondary structure of plus-strand RNAs. Some vdsiRNAs are also generated from the negative strand of the central part, indicating they may be processed from the secondary structure of the negative-strand viroid genomic RNA (Itaya et al., 2007). *Citrus exocortis viroid* (CEVd) replicates in the nucleus and mainly generates 5′-phosphorylated and 3′-methylated vdsiRNAs with positive polarity. Most CEVd vdsiRNAs are located within the right-end domain, suggesting that structured RNA is the main substrate of DCL enzymes (Martin et al., 2007). *Avocado sunblotch viroid* (ASBVd), *Peach latent mosaic viroid* (PLMVd), and *Chrysanthemum chlorotic mottle viroid* (CChMVd) are three viroids that replicate in the chloroplast. CChMVd and PLMVd generate vdsiRNAs from both polarities (de Alba et al., 2002; St-Pierre et al., 2009). ASBVd also generates vdsiRNAs in leaves displaying bleached symptoms (Markarian et al., 2004). Thus, both *Pospiviroidae* and *Avsunviroidae* viroid families can produce vdsiRNAs in plants (Ding and Itaya, 2007; Ding, 2009; Hammann and Steger, 2012). The fact that vdsiRNAs can be generated from both the positive and the negative strand of the viroid genome with strand and position bias indicates that vdsiRNAs are predominately processed from the secondary structure of the viroid genomic RNAs. However, it is important to point out that the discoveries of vdsiRNAs may be biased due to the current methods for sRNA cloning.

dsRNA inducers are processed by plant DCL proteins to generate sRNAs. *Arabidopsis* encodes four DCL proteins that generate different sRNAs: DCL1 processes hairpin pri-miRNAs and pre-miRNAs into 21-nt miRNAs; DCL3, DCL4, and DCL2 process long dsRNAs into 24-nt hc-siRNAs, 21-nt siRNAs, and 22-nt siRNAs, respectively. For RNA viruses and viroids, the perfectly paired dsRNA intermediate replication form and the hairpin structure of the single genomic RNA are predominant dsRNA inducers. Indeed, DCL4, DCL2, and DCL3 process ssRNA viruses (e.g., CMV, TuMV, and TCV) into 21-nt, 22-nt and 24-nt vsiRNAs, respectively (Bouche et al., 2006; Deleris et al., 2006; Fusaro et al., 2006; Diaz-Pendon et al., 2007; Garcia-Ruiz et al., 2010). The newly emerging systemic leaves of PSTVd-infected plants only accumulate shorter (21–22-nt) vdsiRNAs, while the older leaves contain both shorter and longer (24-nt) vdsiRNAs (Machida et al., 2007; Schwind et al., 2009). Similar vdsiRNA accumulation patterns are also present in *Hop stunt viroid* (HSVd)- and *Hop latent viroid* (HLVd)-infected plants. However, very little is known about the biogenesis of vdsiRNAs. 21-nt, 22-nt, and 24-nt vsiRNAs also accumulate in plants infected with *Cabbage Leaf Curl Virus* (CalCuV), *Beet curly top virus* (BCTV), and *Pepper golden mosaic virus* (PepGMV), which are all ssDNA viruses, and CaMV, a dsDNA virus (Blevins et al., 2006; Rodriguez-Negrete et al., 2009; Raja et al., 2014). 24-nt vsiRNAs are the predominant vsiRNAs produced from DNA viruses. DCL3, DCL4, and DCL2 are required for the accumulation of 24-nt, 21-nt, and 22-nt CalCuV vsiRNAs, respectively. DCL3 and DCL4 are also responsible for accumulation of 24- and 21-nt vsiRNAs derived from BCTV, respectively (Raja et al., 2014). Although the hairpin structure of viral or viroid genomes is one of the main sources of vsiRNA and vdsiRNAs, DCL1-dominant hairpin processing is not involved in vsiRNA and vdsiRNAs accumulation or anti-RNA-viral resistance. However, DCL1, but not DCL4, is required for the accumulation of 21-nt vsiRNAs from CaMV (Blevins et al., 2006).

After the initial processing of dsRNA inducers, the antiviral and antiviroid signals are amplified by host RDRs. *Arabidopsis* encodes six RDRs, among which the function of RDR1, RDR2, and RDR6 have been well studied. RDR1 is induced by salicyclic acid (SA) treatment and TMV infection in tobacco and *Arabidopsis*, and a mutation in RDR1 permits efficient multiplication of ssRNA viruses (Xie et al., 2001; Yu et al., 2003; Yang et al., 2004). Furthermore, the *Arabidopsis rdr6* mutant is more susceptible to infection with ssRNA and ssDNA viruses (Dalmay et al., 2000; Mourrain et al., 2000; Dalmay et al., 2001; Muangsan et al., 2004). Infection with RSV (a negative ssRNA virus) and RDV (a dsRNA virus) decreases the expression of rice RDR6. Down-regulation of rice RDR6 by antisense transformation results in increased susceptibility to RDV (Jiang L. et al., 2012; Hong et al., 2015). Both RDR1 and RDR6 are required for secondary CMV vsiRNA production in *Arabidopsis*: RDR1 is required for the production of vsiRNAs from 5′-terminal ends of the genome, while RDR6 is required for the production of vsiRNAs from the 3′-terminal ends (Wang et al., 2010). However, expression of *Nicotiana tabacum* RDR1 in *N. benthamiana* plants (which do not encode RDR1) showed that RDR1 suppresses RNA silencing mediated by RDR6 and enhances viral infection in transgenic plants (Ying et al., 2010). In addition, accumulation of HSVd and PSTVd genomic RNAs was higher in RDR6-silenced plants, indicating that RDRs also contribute to anti-viroid resistance (Gomez et al., 2008; Di Serio et al., 2010). Systematic analysis via profiling of vsiRNAs and vdsiRNAs in pathogen infected plants have revealed that sRNAs processed from pathogen genomic RNAs indeed decrease in *rdr* knock-out mutants or RDR-silenced plants (Gomez et al., 2008; Di Serio et al., 2010; Garcia-Ruiz et al., 2010; Wang et al., 2010; Hong et al., 2015). The decreased accumulation of vsiRNAs and vdsiRNAs and increased susceptibility of *rdr* mutant plants demonstrate the anti-viral/viroid role of RDRs. Although RDR2 is responsible for 24-nt hc-siRNA accumulation, mutation in *RDR2* has little or no effect on the accumulation of vsiRNAs of DNA viruses CalCuV and CaMV (Blevins et al., 2006). Tomato *Ty-1* and *Ty-3* are alleles of the same gene that encodes RDRs with sequence similarity to *Arabidopsis* RDR3, RDR4, and RDR5. They are TYLCV resistance genes, and susceptible tomato lines without these loci produce lower levels of TYLCV vsiRNAs and accumulate higher viral titers (Verlaan et al., 2013; Butterbach et al., 2014). However, in *Arabidopsis*, the antiviral functions of RDR3, RDR4, and RDR5 have not yet been uncovered. Therefore, the function of RDR2 and other RDRs in host-virus/viroid interactions needs to be further explored.

After processing and amplification, vsiRNAs and vdsiRNAs are loaded into AGO proteins to inhibit the replication and movement of viruses and viroids. AGO1 (Morel et al., 2002; Zhang et al., 2006), AGO2 (Takeda et al., 2008; Harvey et al., 2011; Jaubert et al., 2011; Wang X.B. et al., 2011), AGO3 (Schuck et al., 2013), AGO5 (Takeda et al., 2008), AGO7 (Qu et al., 2008), and AGO10 (Garcia-Ruiz et al., 2015) have been shown to bind vsiRNAs or be involved in anti-viral RNA silencing pathways. Recovery from infection with a DNA virus requires the function of host AGO4 (Raja et al., 2008). Thus, the 24-nt vsiRNAs of DNA viruses may associate with AGO4 to methylate the viral genome. A mutant defective in DRB3, a double-stranded RNA binding protein that interacts with DCL3 and AGO4, displays lower methylation of the viral DNA genome and increased hyper susceptibility to germinivirus, further demonstrating the function of the DCL3-AGO4 RdDM pathway in resistance against DNA viruses (Raja et al., 2014). Furthermore, AGO18, a novel AGO that is conserved in monocot plants, is induced by RSV and required for rice antiviral resistance (Wu et al., 2015). In regards to the vdsiRNAs, the 21-nt and 22-nt vdsiRNAs are predominately loaded into AGO1, AGO2, and AGO3 (Minoia et al., 2014); the 24-nt vdsiRNAs are loaded into AGO4, AGO5, and AGO9; while AGO6, AGO7, and AGO10 do not bind vdsiRNAs (Minoia et al., 2014). However, the anti-viroid function of these AGOs needs to be further determined.

Although vdsiRNAs processed by DCLs are loaded into plant AGOs, their regulation of viroid genomes is not well known. PSTVd, CEVd, and CChMVd in plants can be silenced by transgenic dsRNAs or co-inoculated dsRNAs. This silencing is sequence-specific, temperature-dependent and, in some cases, dose-dependent (Vogt et al., 2004; Carbonell et al., 2008; Schwind et al., 2009). However, further studies indicate that viroids may have evolved a mechanism to avoid the silencing of sRNA. Dr. Biao Ding’s group found that PSTVd replicates easily in infected plants even with the present of high accumulation of vdsiRNAs (Itaya et al., 2001). Studies on PSTVd and HSVd show that the circular genome of the viroid is resistant to RNA silencing (Wang et al., 2004; Gomez and Pallas, 2007). A possible explanation is the structured viroid RNA can be processed into active vdsiRNAs, but the viroid RNA is resistant to RISC-mediated degradation due to its secondary structure (Itaya et al., 2007).

## Pathogen sRNAs Regulate Pathogen Gene Expression to Increase Virulence

During plant–microbial pathogen interaction, host miRNAs and siRNAs play a role in modulating host immunity while some sRNAs derived from pathogens can decrease host defense or increase pathogen virulence. Fungi, oomycetes, bacteria, viruses, viroids, and satellite RNAs all produce sRNAs that are either similar to or distinct from plant sRNAs (**Figures 1B**, **2B**, **3B**, and **4B**). During the counter-defense response, these pathogen sRNAs facilitate infection by adjusting pathogen gene expression to increase virulence.

Fungi and oomycetes encode siRNAs that are mainly derived from transposons, inverted, tandem, or other repeat regions, and effector coding regions. These sRNAs display diverse biogenesis pathways, and some require typical RNA silencing components, such as DCLs, AGOs, and RDRs for accumulation (Murata et al., 2007; Lee et al., 2010; Jiang N. et al., 2012; Fahlgren et al., 2013; Qutob et al., 2013; Raman et al., 2013; Weiberg et al., 2013). sRNAs in fungal pathogens have been shown to mediate pathogenic virulence by traveling into host cells and silencing host genes (**Figure 2B**) (Weiberg et al., 2013). Although there is indirect evidence that links sRNAs to pathogen virulence, the function of sRNAs in pathogen cells has not been well studied. The differential accumulation of *M. oryzae* sRNAs in vegetative and specialized-infection tissues suggests that sRNAs in *M. oryzae* may be involved in growth, development, and virulence (Nunes et al., 2011). Moreover, sRNA profiling of *M. oryzae* identified a set of genes that are transcriptionally regulated by sRNAs. One of these is *ACE1*, a known avirulence gene that has increased expression in the *dcl1* mutant (Raman et al., 2013). The sRNAs in three *Phytophthora* species, *P. infestans*, *P. sojae* and *Phytophthora ramorum*, were analyzed, and they were predominantly 21-nt and 25-nt long (Fahlgren et al., 2013). The 21-nt sRNAs were found to be derived from gene families including Crinkler (CRN) effectors and type III fibronectins. Some of these 21-nt sRNAs are predicted to target amino acid/auxin permeases, but their exact functions are still unknown (Fahlgren et al., 2013). sRNAs generated from RxLR and CRN effectors loci were also identified. The expression levels of these effectors and the sRNAs, vary in *P. infestans* strains that differ in virulence, suggesting that these sRNAs may affect the accumulation of effectors, thus alter the virulence (Vetukuri et al., 2012). Some sRNAs map to the tRNA loci of fungi and oomycetes (Nunes et al., 2011; Asman et al., 2014). The biogenesis of these sRNAs requires pathogen DCLs and AGOs. The accumulations of these sRNAs are significantly changed during the infection progress, which suggests that these sRNAs may function in pathogen-host interaction. Moreover, recent study have identified sRNAs associated with *P. infestans* AGO proteins (Asman et al., 2016). PiAGO1-associated 20–22 nt sRNAs, were generated from genes encoding host cell death-inducing CRN effectors, while 24–26 nt sRNAs, which bound to PiAGO4, were derived mainly from Helitron, Crypton, PiggyBac and Copia transposons. The essential role of PiAGO1 in gene regulation, together with its associated sRNAs, which derived from CRN gene family, implicating 20–22 nt sRNAs may bind to AGO1 to regulate the expression of genes in CRN family and subsequently mediate the pathogen virulence (Asman et al., 2016). In addition, sRNAs that are derived from the effector regions can transgenerationally alter the virulence of the pathogen. Avirulence (Avr) gene *Avr3a* of *P. sojae* encodes an effector protein that can be detected by the host *R* gene. The expression of *Avr3a* gene in *P. sojae* attenuated the virulence of plants carrying the *R* gene *Rps*3a. Qutob et al. (2013) observed non-Mendelian inheritance of transgenerational gene silencing of *Avr3a* and gain of virulence in soybean plants. Meanwhile, increased accumulation of 25-nt sRNAs was seen in gene-silenced strains but not in strains with *Avr3a* mRNA, indicating there is sRNA-associated transgenerational gene silencing (Qutob et al., 2013).

Until now bacteria have not been found to encode typical sRNAs as plants, but they produce 50- to 300-nt non-coding sRNAs (ncRNAs) that regulate the expression of target mRNAs through imperfect base-pairing of short regions (10- to 25-nt) (**Figure 1B**) (Altuvia, 2007; Weiberg et al., 2014). There is an emerging body of evidence suggesting that ncRNAs are involved in bacterial virulence. Bacterial lipoprotein (BLP) triggers cell activation and host defense through toll-like receptors (TLRs). CRISPR-Cas-associated sRNAs from *Francisella novicida* guide the Cas9 protein to suppress BLP, which subsequently facilitates evasion of TLR2 (Sampson et al., 2013). The Cas9 system acting with a small, CRISPR/Cas-associated RNA (scaRNA) also controls virulence of *Francisella tularensis* (Sampson et al., 2013). However, a direct link between the Cas system and plant bacterial pathogenesis has not yet been found. Genome-wide transcriptome analysis has identified 16 intergenic sRNAs and seven *cis*-encoded antisense sRNAs in the plant pathogen *Xanthomonas campestris* pv. *vesicatoria* (Xcv) (Schmidtke et al., 2012). The expression of half of these intergenic sRNAs is controlled by components of the type III secretion system, and some are involved in virulence. The deletion of sX12 delays the development of disease symptoms and HR in pepper plants (Schmidtke et al., 2012). The 115-nt sRNA sX13 regulates 63 genes, which are involved in signal transduction, motility, transcriptional and posttranscriptional regulation, and virulence. Deletion of *sX13* strongly delayed development of disease symptoms in susceptible and resistant pepper plants (Schmidtke et al., 2013). However, the function of sX13 is not dependent on Hfq, a hexameric RNA-binding protein that globally interacts with sRNAs to post-transcriptionally regulate gene expression and virulence traits in many animal and plant pathogenic bacteria. Hfq can bind up to 100 sRNAs in *Salmonella* (Chao and Vogel, 2010). Hfp-dependent sRNAs in *Erwinia amylovora* were also identified, and 40 of them were found to associate with Hfq. sRNAs ArcZ, RmaA, and OmrAB all contribute to virulence by positively modulating type III secretion system attachment, amylovoran production, and motility (Zeng et al., 2013; Zeng and Sundin, 2014).

## Pathogens Encode sRNAs Targeting Host Genes to Improve Virulence

Another strategy of pathogens to counteract host defenses is the production of sRNAs that target host genes to decrease host immune responses. Viroids do not code any protein or peptide and yet are able to replicate, travel cell-to-cell and long distance through phloem, resist plant defense responses, and cause disease in certain hosts (Ding and Itaya, 2007; Ding, 2009). For a long time, the question of how viroids produce disease symptoms without any open reading frames has intrigued scientists. Early studies focus on explaining the molecular mechanism of viroid pathogenesis by determining the interaction of genomic RNA of viroid with host factors, including host proteins or nucleic acids (Navarro et al., 2012a,b). While a few proteins or RNAs were determined to interact with viroid RNA, their roles in viroid pathogenesis is largely inclusive. In recent years, the new hypothesis that viroids cause disease symptoms by producing sRNAs to target host genes was raised and there are many studies supporting this hypothesis (**Figure 4B**). Over-expression of PSTVd hairpin RNA, which produces sRNAs, results in similar phenotypes as PSTVd infection, suggesting that PSTVd may cause disease symptoms by sRNA-mediated silencing (Wang et al., 2004). Large-scale sequencing uncovered that two genes involved in gibberellin or jasmonic acid biosynthesis contain binding sites for PSTVd vdsiRNAs (Wang et al., 2011b). Moreover, DCL4, which should reduce PSTVd levels by slice or dice its genome RNA to produce vdsiRNA, seems to benefit the accumulation of PSTVd (Dadami et al., 2013). Expression of an artificial miRNA containing the sequence of the PSTVd virulence modulating region down-regulates the expression of a *Nicotiana* soluble inorganic pyrophosphatase (siPPase) gene and leads to a PSTVd infection phenotype (Eamens et al., 2014). In addition, a recent study showed that single vdsiRNA is able to silence multiple host mRNAs. vdsiRNAs derived from PSTVd can target two callose synthase genes, CalS11-like and CalS12-like, which are essential for the formation of callose. The efficiency of suppression depends on the viroid variants and the target gene (Adkar-Purushothama et al., 2015). PLMVd is a chloroplast-replicating viroid and an insertion of a 12- to 13-nt fragment inhibits chloroplast development (Rodio et al., 2007). Further study uncovered that in *Prunus persica*, two vdsiRNAs containing the insertion sequence target the chloroplast heat shock protein 90 (HSP90) and triggers signal transduction that eventually leads to viroid disease symptoms (Navarro et al., 2012a). A single U257A change in the PSTVd central conserved region also strongly increases PSTVd virulence by restricting host cell expansion. The lethal phenotype of PSTVd is correlated with the down-regulation of *LeExp2* gene expression (Qi and Ding, 2003). It is not clear whether the U257A mutation also produces a novel sRNA that targets some essential host genes that is critical for cell expansion and *LeExp2* expression. Furthermore, upon viroid infection, vdsiRNAs generated by *Tomato planta macho viroid* (TPMVd) targets and slices the SolWD40 gene, the function of which is unknown (Avina-Padilla et al., 2015). Although HSVd genomic RNA is higher in RDR6-silenced plants, the viroid-induced symptoms are absent. Meanwhile, HSVd vdsiRNA accumulation is decreased in RDR6-silenced plants, suggesting that the symptoms of HSVd is dependent on vdsiRNAs (Gomez et al., 2008). The symptom severity of CEVd is also correlated with the level of vdsiRNAs but not the viroid genome level, further supporting that vdsiRNAs are not simply by-pass products of anti-viroid RNA silencing reactions, but they have a purpose in producing disease symptoms karian (Markarian et al., 2004). In addition, there are some evidence that link viroid infection to transcriptional gene silencing (TGS). Wassenegger et al. (1994) discovered that PSTVd cognate DNA sequences were methylated in PTSVD-expressing transgenic tobacco plant, while the T-DNA and the genomic plant DNA remained unaltered. Further studies also demonstrate the correlations between viroid infection and host genes transcriptional alteration. For instance, cucumbers infected with HSVd accumulate high levels of sRNAs derived from ribosomal transcripts, as well as ribosomal RNA (rRNA) precursors. This was caused by altered DNA methylation in the promoter region of rRNA genes, resulting in demethylation and transcriptional reactivation of normally inactive rRNA genes (Martinez et al., 2014). *N. benthamiana* carrying an HSVd dimeric sequence develops similar phenotype to HSVd-infected plants (Gomez et al., 2008). This plant also accumulates high levels of sRNAs derived from ribosomal transcripts along with a decrease in rDNA methylation, suggesting that this may be a general phenomenon (Castellano et al., 2015). However, the correlation between sRNA accumulation and DNA methylation needs to be further determined.

It is noteworthy that although some studies suggest that symptoms produced by viroids in plants are associated with vdsiRNAs and the RNA silencing machinery, there is no uniform correlation between the levels of vdsiRNA and symptoms (Ding and Itaya, 2007; Ding, 2009; Kovalskaya and Hammond, 2014). Moreover, in contrast to early observation that symptoms similar to those of PSTVd infection were developed in some transgenic tomato lines expressing non-infectious PSTVd hairpin RNA (Wang et al., 2004), no disease symptoms were found in other tomato lines, despite the accumulation of PSTVd hairpin-derived siRNA (Schwind et al., 2009). Whether vdsiRNA indeed results in viroid disease symptoms requires further investigation.

Plant viruses are often accompanied with a variety of subviral RNA/DNAs, which have no or little sequence similarity to plant viruses. Most satellite RNAs do not encode proteins but can significantly alter viral disease symptoms (**Figure 3B**) (Collmer and Howell, 1992; Simon et al., 2004). More and more studies indicate that the pathogenicity of satellite RNA/DNA may due to host gene silencing induced by satRNA-derived siRNAs (satsiRNAs). CMV Y satellite (Y-sat) causes a bright yellow mosaic phenotype. Replication of Y-Sat is resistant to RNA silencing, but expression of viral suppressors of RNA silencing (VSR) reduces the disease symptoms (Wang et al., 2004). The hairpin structures of CMV satellite RNA are processed by DCL4 and other DCL proteins to form 21-nt and 22-nt satsiRNAs (Du et al., 2007). Y-sat produces a 22-nt satsiRNA that targets Chll, a key gene involved in chlorophyll synthesis, and cleaves Chll mRNA post-transcriptionally, causing the bright yellow mosaic phenotype. Transformation of *N. tabacum* with a silencing-resistant version of Chll greatly reduces the Y-Sat symptoms (Shimura et al., 2011; Smith et al., 2011). satsiR-12, another satsiRNA generated from SD-CMV satellite RNA, targets the upstream region of the CMV 3′ UTR for slicing. satsiR-12 is loaded into AGO1/2/5 for RDR6-mediated regulation, which can be suppressed by 2b encoded by CMV (Zhu et al., 2011). However, the accumulation of 2b coding subgenomic RNA, RNA4A and 2b proteins is also reduced by SD-CMV satellite RNA, which attenuates the D-CMV yellow symptom in *N. benthamiana* (Hou et al., 2011). TCV is often accompanied with a single strand satellite RNA, satC, that is composed of the 3′ end of TCV helper virus. The presence of satC represses the accumulation of TCV genomic RNA and virion, which leads to increased levels of free CP proteins. CP is a VSR encoded by TCV that targets the DCL2/4 silencing pathway and suppresses satC accumulation. The satC-mediated enhancement of free CP proteins then increases the symptoms of TCV (Zhang and Simon, 2003; Manfre and Simon, 2008). Thus, sRNAs generated from satellite RNAs produce species-specific disease symptoms by targeting host genes or viral genomes. On the other hand, in the presence of SD-CMV satellite RNA, the infection of CMV-Δ2b lead to high accumulation of satsiRNA, while the accumulation of CMV siRNA was reduced. Thus, the dice and slice of host RNA silencing machinery on SD-CMV satellite RNA may decrease its efficiency on CMV RNAs (Hou et al., 2011). DNA β satellites are circular ssDNA that associate with many monopartit begomoviruses and are essential for viral disease symptoms (Briddon et al., 2001; Jose and Usha, 2003; Cui et al., 2004). The ßC1 protein encoded by DNA β satellite is a VSR that suppresses methylation-mediated TGS and RDR6-mediated PTGS through the interaction of *S*-adenosyl homocysteine hydrolase (SAHH) and rgs-CaM, which will be discussed later (Cui et al., 2005; Yang et al., 2011b; Li et al., 2014). Thus, viral satellite RNA/DNA can alter the symptoms caused by the helper virus with different sRNA related mechanisms.

Upon infection with a virus, host plants process viral genomic or transcript RNAs into vsiRNAs and load them into RISC complexes to inhibit the amplification and movement of the virus. However, depending on the similarity of vsiRNA-target gene and host genes, some vsiRNAs can target host genes, which subsequently increase viral pathogenicity (**Figure 3B**). Deep sequencing and bioinformatics studies indicate that 16 TMV vsiRNAs potentially target *Arabidopsis* genes. Two of these vsiRNAs target and slice transcripts of a polyadenylation specificity factor and an unknown protein similar to translocon-associated protein alpha. The slicing of these two genes only happens upon TMV infection, revealing that they are real vsiRNA targets (Qi et al., 2009). Dozens of *Zea mays* genes are predicted targets of vsiRNAs encoded by *Sugarcane mosaic virus* (SCMV). Some vsiRNA targets that contribute to biotic/abiotic stress responses and ribosome biogenesis are down-regulated upon SCMV infection (Xia et al., 2014). In addition, vsiRNAs originating from the leader region of CaMV 35S RNA were found to increase the accumulation of CaMV. Like other vsiRNAs, these leader-derived vsiRNAs are DCL-dependent and subsequently loaded into AGO1 (Blevins et al., 2011). These vsiRNAs may also facilitate CaMV accumulation by suppressing *Arabidopsis* gene expression. RSV infection causes plant stunting, chlorosis, and other symptoms. A recent study showed that vsiRNAs can be generated from RSV RNA4, and further targeting host gene *eIF4A*. The infection of RSV down-regulated eIF4A expression. Interestingly, eIF4A suppression by artificial miRNAs leads to rice leaf-twisting and stunting (Shi et al., 2016). Thus, vsiRNAs can directly cause virus pathogenicity, as with vdsiRNAs. Nine chloroplast-related genes (ChRGs) are also down-regulated upon RSV infection and silencing them with artificial miRNAs causes plant chlorosis symptoms, similar to viral infection. However, whether the down-regulation of ChRGs upon RSV infection is also mediated by RSV vsiRNAs need to be further studied (Xia et al., 2014). In contrast to siRNAs, there are relatively few studies done on virus-encoded miRNAs in plants. Studies on *Sugarcane streak mosaic virus* (SCSMV) and *Hibiscus chlorotic ringspot viru*s (HCRSV) suggest the existence of virus-encoded miRNAs that may target plant genes, but their detailed functions remain unknown (Gao et al., 2012; Viswanathan et al., 2014).

Fungi, omycetes, and bacteria that localize in the intercellular region in the early infection stages can deliver pathogen sRNAs into plant cells to target host genes as counter-defense. Infecting *Arabidopsis* and *Solanum lycopersicum* with a destructive fungal plant pathogen, *Botrytis cinerea*, results in the presence of a set of *B. cinerea* sRNAs (Bc-sRNAs) in both plants. Among these sRNAs, 73 Bc-sRNAs are able to target host genes in both *Arabidopsis* and *S. lycopersicum*. These Bc-sRNAs are processed by fungi DCLs and loaded into a host AGO1 protein to slice host targets. A mutation in *Arabidopsis AGO1* reduces the susceptibility of the plant to *B. cinerea*, and a mutation in *B. cinerea DCLs* decreases fungi pathogenicity. Multiple Bc-sRNA target genes were identified, including *Arabidopsis* mitogen-activated protein kinase genes *MPK1* and *MPK2*, a cell wall-associated kinase (*WAK*), a peroxiredoxin (*PRXIIF*), and the tomato MPK-kinase kinase 4 (*MAPKKK4*). Suppression of these genes increases the disease susceptibility of the plant (Weiberg et al., 2013). This is the first study showing that sRNAs from a eukaryotic pathogen mediate pathogen virulence using host RNA silencing machinery; however, it is still unclear how these fungal siRNAs are delivered into plant cells. Pathogen sRNAs have been shown to be delivered into animal cells though RNA transporters. Two membrane-associated RNA transporters, systemic RNAi defective-1 (SID1) and SID2, were identified in *C. elegans* (Shih and Hunter, 2011; McEwan et al., 2012). However, no membrane-associated RNA transporters have yet been identified in plants.

RNA silencing inhibits the infection, replication, and movement of same viruses at different steps. Thus, pathogens also encode RNA silencing suppressors to decrease the accumulation of sRNAs or inhibit the function of sRNAs (Qi et al., 2004). Many VSRs are viral pathogenicity determinants, indicating that the suppression function is important for pathogenicity. Some VSRs bind viral dsRNA or vsiRNAs and decrease the number of functional sRNAs targeting viral genomes. Other VSRs directly or indirectly target RNA silencing pathway components such as DCLs, RDRs, and AGOs to inhibit the accumulation and function of endogenous miRNAs and siRNAs, thus increasing the severity of infection symptoms (Csorba et al., 2015). It is believed that plant viruses encode multiple VSRs or a multi-functioning VSRs and express them in host cells to counteract host defenses. For examples, the AL2 VSRs encoded by DNA virus CalCuV silences both transcription-dependent PTGS (transcription activation with the interaction with WEL1 and rgs-CaM) and transcription-independent PTGS (ADK inactivation with the interaction with ADK) (Wang et al., 2003; Trinks et al., 2005; Yong Chung et al., 2014). A recent study uncovered that AL2 also reverses TGS by a transcription-activation- and ADK inactivation-independent mechanism (Jackel et al., 2015). Pns10 encoded by RDV can not only bind siRNAs but also down-regulate RDR6 expression to suppress RNA silencing for viral replication and movement (Cao et al., 2005; Ren et al., 2010). While the replication of RNA viruses is suppressed by PTGS, the replication of DNA viruses is inhibited by both PTGS and TGS (Raja et al., 2008). The function of DNA VSR in the accumulation of 24-nt TGS siRNAs and RdDM pathway components has also been determined recently: C2 (also known as AL2 or AC2) inhibits the ADK function and attenuates the degradation of SAMDC1; C4 down-regulates the accumulation of MET1 but not CMT3; Rep represses the expression of MET1 and CMT3; V2 of TYLCV and AC5 of *Mungbean yellow mosaic India virus* (MYMIV) decreases the methylation of transgenic and endogenous loci by an unknown function; betasatellite βC1 inhibits the activity of SAHH (Wang et al., 2003, 2014; Yang et al., 2011b; Zhang Z. et al., 2011; Rodriguez-Negrete et al., 2013; Li et al., 2015).

Some bacteria and oomycetes also deliver effector proteins into host cells to suppress RNA silencing. Although miR393 is induced upon *Pst* DC3000 infection, the AvrPtoB effector specifically represses the induction of miR393 at the transcriptional level. AvrPto also reduces miR393 accumulation. However, the accumulation of pri-miR393 is not changed in transgenic plants, which indicates that AvrPto may post-transcriptionally down-regulate the processing of miR393 (Navarro et al., 2008). Oomycete *P. sojae* encodes two RNA silencing suppressors: PSR1 down-regulates the accumulation of both host miRNAs and siRNAs, while PSR2 specifically decreases the accumulation of host siRNAs (Qiao et al., 2013). Both of them are effector proteins and their over-expression enhances the infection of *Phytophthora* and viruses. PSR1 interacts with PINP1, a RNA helicase that regulates the accumulation of both miRNAs and siRNAs. The over-expression of *PSR1* or the down-regulation of *PINP1* impairs the localization of the DCL1 protein complex (Qiao et al., 2015). Another PSR2 protein encoded by *P. infestans* can also suppress RNA silencing and enhance the plant susceptibility to *Phytophthora (*Xiong et al., 2014*)*. Thus, the RNA silencing suppressors encoded by oomycetes might be a general counter-defense mechanism. It will be interesting to see whether fungi also deliver effector proteins to inhibit host resistance.

## Conclusion

There is an increasing amount of evidence that shows communication occurs between plants and different pathogens via sRNAs. The importance of sRNAs in regulating plant immunity and pathogen virulence allows scientists to utilize and manipulate RNA silencing machinery to improve plant immunity, impair pathogen virulence, and thus increase crop production. RNAi technology has been employed to manipulate plant metabolites, develop plants with improved resistance to environment stresses, and engineer plants to defend against pathogen infections (Koch and Kogel, 2014). In plants, expression of pathogen dsRNAs is widely used for plant resistance to viruses that replicate in plant cells. The different roles of sRNAs have also been demonstrated in anti-fungal, anti-insect, anti-nematode resistance, pointing to the existence of cross-kingdom RNA silencing (Baum et al., 2007; Mao et al., 2007; Nowara et al., 2010; Ibrahim et al., 2011; Koch et al., 2013; Panwar et al., 2013). However, RNA silencing is a complicated system, and there are two sides to the coin. For instance, while plants utilize vsiRNAs to silence viral RNA as a defense strategy, vsiRNAs can also target plant mRNAs to promote viral virulence. The never-ending arms race drives the co-evolution of pathogen and hosts, resulting in the variety of sRNAs and RNAi components. To utilize RNA silencing machinery, further investigation is required to explore this complicated and fascinating sRNA world.

## Author Contributions

JH wrote the introduction, the summary of sRNA in plant and virus. MY wrote the summary of sRNA in bacteria and fungi. Both authors contributed to the figures and figure legend. XZ and LL supervised and complemented the writing.

## Conflict of Interest Statement

The authors declare that the research was conducted in the absence of any commercial or financial relationships that could be construed as a potential conflict of interest.
